# Tissue damaging toxins in snake venoms: mechanisms of action, pathophysiology and treatment strategies

**DOI:** 10.1038/s42003-024-06019-6

**Published:** 2024-03-22

**Authors:** Mátyás A. Bittenbinder, Jory van Thiel, Fernanda C. Cardoso, Nicholas R. Casewell, José-María Gutiérrez, Jeroen Kool, Freek J. Vonk

**Affiliations:** 1 Naturalis Biodiversity Center, 2333 CR Leiden, The Netherlands; 2 AIMMS, Division of BioAnalytical Chemistry, Department of Chemistry and Pharmaceutical Sciences, Faculty of Sciences, Vrije Universiteit Amsterdam, De Boelelaan 1085, 1081HV Amsterdam, The Netherlands; 3 Centre for Analytical Sciences Amsterdam (CASA), 1098 XH Amsterdam, The Netherlands; 4 Centre for Snakebite Research & Interventions, Liverpool School of Tropical Medicine, Pembroke Place, Liverpool, L3 5QA Liverpool, United Kingdom; 5 Institute of Biology Leiden, Leiden University, Sylviusweg 72, 2333 BE Leiden, The Netherlands; 6 Institute for Molecular Bioscience, The University of Queensland, St Lucia, Brisbane, Queensland Australia; 7 Centre for Innovations in Peptide and Protein Science, The University of Queensland, St Lucia, Brisbane, Queensland Australia; 8 Instituto Clodomiro Picado, Facultad de Microbiología, Universidad de Costa Rica, San José, 11501 Costa Rica; 9 Present Address: Howard Hughes Medical Institute and Department of Biology, University of Maryland, College Park, MD 20742 USA

**Keywords:** Chronic inflammation, Apoptosis

## Abstract

Snakebite envenoming is an important public health issue responsible for mortality and severe morbidity. Where mortality is mainly caused by venom toxins that induce cardiovascular disturbances, neurotoxicity, and acute kidney injury, morbidity is caused by toxins that directly or indirectly destroy cells and degrade the extracellular matrix. These are referred to as ‘tissue-damaging toxins’ and have previously been classified in various ways, most of which are based on the tissues being affected (e.g., cardiotoxins, myotoxins). This categorisation, however, is primarily phenomenological and not mechanistic. In this review, we propose an alternative way of classifying cytotoxins based on their mechanistic effects rather than using a description that is organ- or tissue-based. The mechanisms of toxin-induced tissue damage and their clinical implications are discussed. This review contributes to our understanding of fundamental biological processes associated with snakebite envenoming, which may pave the way for a knowledge-based search for novel therapeutic options.

## Abstract (web summary)

The snake venom toxins responsible for tissue damage, their mechanisms of action and pathological effects are reviewed, together with the search of novel therapeutic alternatives to abrogate their effects

## Introduction

Snakebite envenoming is a global health challenge and a neglected tropical disease. It accounts for 2.5 million victims each year, and annual mortality estimates range between 81.000 – 138.000^1^. Snake venoms cause many local and systemic effects in humans, with some being life-threatening while others being permanently debilitating^1–3^. Without early and effective antivenom treatment, morbidity following snakebite can cause permanent disability and disfigurement^4,5^. Snakebite hotspots are found in sub-Saharan Africa, South and Southeast Asia, and Latin America^1,6^. Human snakebite victims are often young male agricultural workers, severely affecting their working abilities and daily activities. Snakebites often leave people with permanent physical and psychological disabilities, significantly affecting their lives in many ways^5^. This causes a substantial socioeconomic impact on families and local economies^7^.

Snake venom is a mixture of peptides and proteins that evolved to disrupt physiological pathways in a prey item but also severely affects humans during defensive snakebites. The two major families of medically important venomous snakes are the elapids (Elapidae, e.g., cobras, mambas, kraits, and coral snakes) and viperids (Viperidae, e.g., vipers and pit vipers). Other families of venomous snakes include Colubridae, Natricidae and Dipsadidae (i.e., non-front-fanged snakes) and Lamprophiidae (e.g., stiletto snakes)^1,8^. However, these are rarely responsible for causing life-threatening human envenomings. Clinical effects of envenoming can be broadly divided into three main pathologies and pathophysiologies: neurotoxicity, haemotoxicity and tissue-damaging effects (which include cytotoxicity, e.g., myotoxicity, and degradation of the extracellular matrix), with some venoms inducing a combination of these.

Neurotoxic effects are caused by toxins affecting synaptic transmission, for example, by hydrolysing phospholipids at the presynapse, acting as antagonists of the cholinergic receptors, or blocking certain ion channels^9–11^. These effects may ultimately result in the impairment of neuromuscular transmission, resulting, among other effects, in respiratory paralysis^3^. Other toxins act in the synaptic cleft by inhibiting acetylcholine esterase, thereby reducing the removal of acetylcholine, causing overstimulation of the muscles, resulting in spasm or fasciculation^11,12^. Toxins responsible for neurotoxic effects are generally divided into two main classes: α-neurotoxins and β-neurotoxins, depending on whether they act post- or pre-synaptically, respectively^10^. The dendrotoxins form a third group of neurotoxins, which belong to the family of Kunitz-type proteinase inhibitors^13,14^.

Haemotoxicity can be considered an umbrella term for many cardiovascular disturbances and haemostatic effects caused by snake venoms. Blood clotting can be affected in such a way that coagulation is either accelerated (i.e., procoagulation) or impaired (i.e., anticoagulation). Toxins that promote coagulation generally affect blood clotting factors by i) activating factor X, prothrombin and other clotting factors, ii) inducing platelet aggregation, or iii) having a thrombin-like (fibrinogenolytic) effect^15,16^. Clinically, procoagulant venom components are responsible for venom-induced consumption coagulopathy associated with incoagulability^15^. Anticoagulant compounds include those that directly inhibit haemostasis by i) modulating platelets, ii) inhibiting platelet aggregation, iii) hydrolysing or binding to phospholipids, which are co-factors for the coagulation cascade, or iv) degrading coagulation factors^15^. In addition to influencing haemostasis, venoms may affect the cardiovascular system by causing local and systemic haemorrhage or by inducing plasma extravasation. Haemorrhage is caused by proteolytic degradation of key components in the basement membrane of the microvasculature, thereby affecting the integrity of the capillary blood vessels. This results in the mechanical weakening of the capillary wall and subsequent extravasation^17–19^. In addition to haemorrhagic toxins, several components in snake venoms induce increments in vascular permeability, thus generating plasma extravasation, which might contribute to hypovolemia and haemodynamic disturbances^20^.

**Fig. 1:**
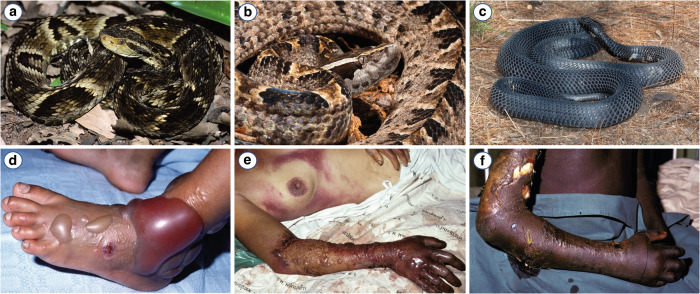
Representative venomous snakes and tissue-damaging effects associated with their envenomings. **a–c** Medically important snake species with tissue-damaging properties in their venoms; (**a**) jararaca (*Bothrops jararaca*); (**b**) Malayan pitviper (*Calloselasma rhodostoma*); (**c**) black-necked spitting cobra (*Naja nigricollis*). **d**–**f** Pathologies caused by cytotoxic snake venoms; (**d**) swelling and blistering following a bite of *B. jararaca*; (**e**) swelling, blistering and necrosis as a result of a bite from *C. rhodostoma*; (**f**) extensive skin- and subcutaneous necrosis following a bite of *N. nigricollis*. Photographs of *B. jararaca* and *C. rhodostoma* courtesy of Wolfgang Wüster; the picture of *N. nigricollis* was taken by Johan Marais (African Snakebite Institute). Photographs of clinical cases by David A. Warrell, published in Gutiérrez et al. ^1^ Nat. Rev. Dis. Primers 3: 17079.

Tissue-damaging effects are the leading cause of snakebite morbidity, including life-long disabilities such as permanent muscle tissue loss, contractures, hypertrophic scars, chronic ulceration, chronic renal disease, ocular damage and other debilitating pathologies (see Fig. 1)^1^. Severe morbidity is conservatively estimated to occur in 400,000 bite victims each year^1^. Despite these morbidity rates, considerably less research has been performed on the tissue-damaging effects than on the neurotoxic and haemotoxic effects of venoms. Toxins with tissue-damaging capabilities can be broadly divided into two main groups based on the way they affect cells (and thus tissues). These include (a) cytotoxins, defined as toxins that are ‘truly’ cytotoxic by directly affecting the viability of cells and (b) extracellular matrix (ECM) degrading enzymes, which, in addition to this action, may be indirectly cytotoxic, meaning that cell death occurs as a secondary effect and not by directly damaging the cells. These cytotoxic components cause a range of pathologies, the most relevant of which are i) local and systemic myonecrosis, ii) dermonecrosis, and iii) acute kidney injury^21–25^. In turn, ECM-degrading enzymes are involved in i) local and systemic haemorrhage, ii) blistering and iii) dermonecrosis^26,27^.

Traditionally, some toxin (sub)classes have been categorised based on the tissues they predominantly affect, e.g., cardiotoxins or myotoxins^21,28^. This classification has limitations as it oversimplifies the complexity of these compounds, as many of them affect several tissues. In addition, owing to its phenomenological nature, this categorisation does not involve the mechanistic aspects of these toxins. Here, we review the molecular mechanisms of venom-induced tissue damage, revealing that snake venoms exert their cytotoxic effects via a number of distinct mechanisms, both direct and indirect. This provides the basis to reclassify the cytotoxin nomenclature based on mechanisms of action rather than on the affected tissue type. By incorporating the fundamental knowledge of the mechanistic pathways of tissue damage, we discuss their clinical impact, along with potential therapeutic options to reduce the severe morbidity of snakebite envenoming. Understanding the mechanisms of action of snake venom toxins that inflict tissue damage may shed light on other diseases involving similar cytotoxic effects.

## A shift in the nomenclature of cytotoxic venom components

**Fig. 2:**
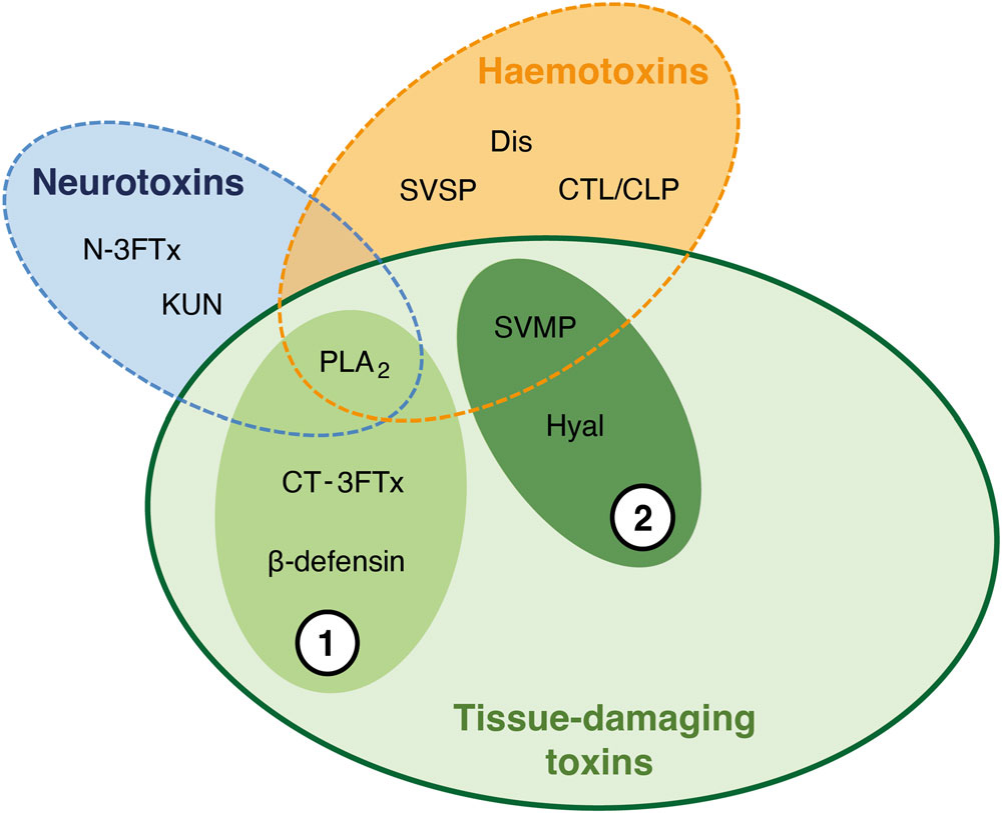
Main types of snake venom toxins and their mechanisms of action. Some toxins have more than one biological effect, thereby creating a multi-layered image. Numbers correspond to two categories of venom-induced tissue damage: (1) direct cytotoxic effects by ‘true’ cytotoxins and (2) degradation of extracellular matrix, which may result in indirect cytotoxic effect. Toxin classes: N-3FTx: neurotoxic three-finger toxins; KUN: Kunitz-type peptides; PLA_2_: phospholipase A_2_s; CT-3FTxs: cytotoxic three-finger toxins; SVMP: snake venom metalloproteinases; Hyal: hyaluronidases; CTL: C-type lectins; CLP: C-type lectin-related proteins; Dis: disintegrins; SVSP: Snake venom serine proteinases. This figure was based on Ray Morgan’s ‘The Venom Interviews’, Part VII, It’s Complicated, 2016.

A thorough understanding of the pathological and pathophysiological effects caused by tissue-damaging toxins is crucial to grasp the complexity of snakebite envenoming and to develop effective therapies for treating venom-induced morbidity. Historically, toxin classes were categorised based on the effects they cause, with a primary division of neurotoxicity, haemotoxicity and tissue-damaging toxicity (see Fig. 2). Some toxin classes are further subdivided based on the tissue type that is affected, such as myotoxins (toxins that target skeletal muscle cells) or cardiotoxins (toxins that target cardiomyocytes). However, this categorisation is inadequate as multiple toxin classes may cause similar pathologies but exert their effects via entirely different molecular mechanisms. Thus, a mechanistic classification instead of a phenomenological one is needed.

The classification based on affected tissue type likely originates from the cell type on which the effect was initially tested. The most striking example can be found in the earliest records of “cardiotoxins”, which were described in the 1940s. Sakar and colleagues described a toxin from cobra venom that is ‘…responsible for the cardiac failure’ and therefore described it as “cardiotoxin”^29,30^. However, studies later showed that the primary effect of these toxins was at the membrane level, leading to a range of effects, including haemolysis, cytolysis of various cell types and muscle fibre depolarisation^31,32^. This led to many alternative names to describe these toxins, such as “cytotoxin”, “direct lytic factor”, “membrane toxin”, and others (see Supplementary Table 1). Although the term “cytotoxin” has now been widely adopted in the literature for this group of toxins, many studies remain using alternative names. Therefore, we propose to use the term “cytotoxic three-finger toxin (3FTx)” as this describes both the basal activity as well as the venom protein family and thus prevents confusion. Interestingly, molecular analysis of so-called cytotoxic 3FTx and cardiotoxins revealed that both ‘groups’ are similar in terms of their primary structure, which suggests that these are not two distinct subclasses, but rather a single group of toxins^33^.

Another example of nomenclature confusion can be found within the cytotoxic phospholipases A_2_ (PLA_2_s), which are often named myotoxins, as they are known to disrupt the plasma membrane of muscle fibres^21^. However, studies on purified myotoxic PLA_2_s on other cell types have proven that the activity spectrum of these toxins is actually much broader than previously thought^34,35^. This underpins the fact that listing these toxins as “myotoxic” is inadequate or at least not mutually exclusive, as these PLA_2_s could be confused with other toxins with myotoxic effects (e.g., small basic myotoxins, metalloproteinases) that belong to a different toxin class and may exert their effects via different mechanisms^26,36–39^. We therefore suggest using the term “cytotoxic PLA_2_s” to describe this specific group of toxins, regardless of which is the primary cellular target.

Although multiple studies have mentioned the limitations of the classification of cytotoxic compounds based on their effects, no decisive term has been suggested to denote this group of toxins. Our suggestion would, therefore, be to start using the term “cytotoxin” for all toxins with direct cytotoxic properties and use clear, distinctive names for categorising the specific toxin classes. Meaning that terms such as “cardiotoxins” would be regarded as “cytotoxic 3FTxs” and “myotoxins” would be considered either “cytotoxic PLA_2_s” or “β-defensin-like toxins”, depending on the toxin class (see Supplementary Table 1).

## A structural-mechanistic classification of venom cytotoxins

**Fig. 3:**
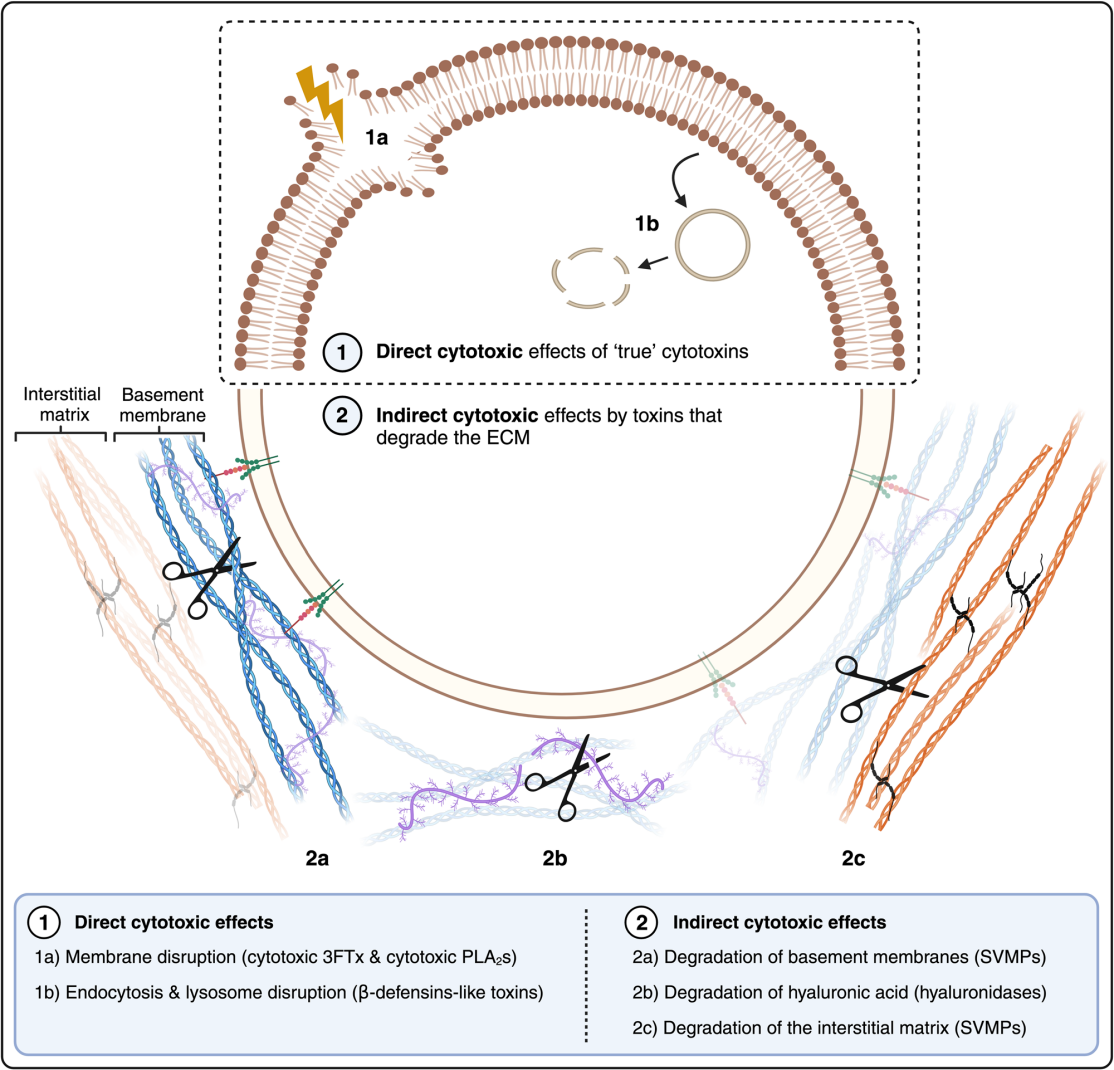
Schematic overview representing the various mechanisms of action of tissue-damaging toxins. (1) Direct cytotoxic effects caused by cytotoxic 3FTxs, cytotoxic PLA2s and β-defensin-like toxins. (2) Degradation of the ECM by SVMPs and hyaluronidases. Degradation of ECM contributes to the diffusion of venom components and can contribute to cellular damage indirectly by affecting the stability of endothelial cells in capillaries and by reducing blood supply as a consequence of haemorrhage, thus generating ischemia. The image was created via www.biorender.com (with permission).

A wide variety of proteins that may cause tissue damage have been characterised in snake venoms. One way to classify these toxins is based on their primary mechanism of action, in which we differentiate between ‘true’ cytotoxins (i.e., toxins that have a direct cytotoxic effect on cells) and toxins that degrade the ECM and may have an indirect cytotoxic effect (e.g., by affecting the ECM which could eventually lead to cell injury) (see Fig. 3).

### Direct cytotoxic effects of ‘true’ cytotoxins

Cell membranes are crucial in maintaining normal cellular functioning. Membranes form an integral part of the cell by separating cellular environments from the plasma membrane to the compartment membranes of the mitochondria, lysosomes, Golgi complex, nuclei, and the endoplasmic reticulum^40^. This could explain why many snake venom toxins deploy their cytotoxic effect by altering membrane integrity, especially the plasma membrane.

### Damage to cell membranes by cytotoxic 3FTxs

The 3FTx superfamily includes small (6-9 kDa), non-enzymatic toxins that have a wide array of biological functions, both as neurotoxins (i.e., short- and long-chain neurotoxins) and cytotoxins (i.e., cytotoxic 3FTxs)^41^. The latter are found exclusively in four related elapid genera, including *Naja*, *Hemachatus, Ophiophagus* and *Aspidelaps*^33^. The exact mechanisms by which cytotoxic 3FTxs exert their effects are not yet fully elucidated, and competing hypotheses exist. These hypotheses include cytotoxicity caused by interactions with plasma membrane components resulting in pore formation and cell damage secondary to lysosome lysis. Cytotoxic 3FTxs differ from non-cytotoxic 3FTxs by their hydrophobic character, with the hydrophobic patch being absent in the neurotoxic members of this family^32,42,43^. The highly conserved hydrophobic core in cytotoxic 3FTxs is thought to interact non-specifically with the hydrophobic structure of the lipid bilayer of cell membranes. Although the exact mechanism has still to be elucidated, it is believed that the cytotoxic 3FTxs exert their effect by forming pores in the cell membrane^42,44,45^. These pores could be formed by cytotoxic 3FTxs alone or by an (oligomeric) association of cytotoxic 3FTxs^45^.

Another mechanism was proposed by Feofanov et al., which included binding to the plasma membrane, followed by internalisation and subsequent transportation to lysosomes. In this event, the plasma membrane permeabilisation is suggested to be a secondary effect following the lysosome rupture^46^. Additionally, it has been proposed that cytotoxic 3FTxs may be involved in the activation of intracellular signalling cascades, activating several cell death pathways^47^. However, further evidence is required to support these mechanisms.

Cytotoxic 3FTxs are known for their synergy with PLA_2_s, which potentiate their cytotoxic effects. This phenomenon was first reported by Condrea et al., who showed a significant increase in erythrocyte lysis with cytotoxic 3FTx and PLA_2_ fractions tested together, compared to separately^48^. This toxin synergy has then been described in numerous studies over the years^33,49–52^. Interestingly, the cytotoxic effects of cytotoxic 3FTxs from cobras can be enhanced by PLA_2_s of distinct species of snakes (as shown for PLA_2_s of several elapids and vipers), suggesting that this synergy is not only restricted to venom PLA_2_s of the same species^51^.

In addition to the cytotoxic effects, this toxin synergy is associated with increased pain levels due to the enhanced effects on sensory neurons. It underlies the evolution of defensive venom spitting in spitting cobras^33^. Evidence suggests that some cytotoxic 3FTxs and PLA_2_s form protein complexes together by homo- or hetero-oligomerisation^45,51^. However, the exact molecular mechanisms that cause cell death, as well as the actual complex formation, remain unknown. Pucca *et al*. proposed two mechanistic models that may explain the synergically potentiated cytotoxic effects of the cytotoxic 3FTx-PLA_2_s complex^51^. First, given that PLA_2_s bind more easily to outer plasma membranes compared to cytotoxic 3FTx, the cytotoxic 3FTx-PLA_2_s complex would have an advantage in binding and thus could synergistically enhance the cytotoxic effects. Another explanation could be that plasma membrane integrity would be affected by PLA_2_s-induced phospholipid hydrolysis, and this would render the membrane more susceptible to the lytic action of cytotoxic 3FTxs. However, more research is needed to elucidate the exact molecular mechanisms of the cytotoxic enhancing effects of this toxin synergy.

### Destabilisation of cell membranes by cytotoxic PLA_2_s through enzymatic and non-enzymatic mechanisms

PLA_2_s are one of the major toxin classes in snake venoms. This toxin superfamily has been extensively studied as they are among the most abundant toxins in viper and elapid snakes^1,53,54^. These small (~13–15 kDa) proteins have a wide toxicological profile that includes neurotoxic, haemotoxic and cytotoxic effects^53,55^. Snake venom PLA_2_s can be divided into two groups based on their structural characteristics. Group I PLA_2_s are found in elapid venoms, whereas PLA_2_s in the viperid family are classified as Group II PLA_2_^55^. While these PLA_2_s share a similar catalytic mechanism, they present distinct structural features. Some group I PLA_2_s are non-toxic, while some are presynaptic neurotoxins, and cytotoxic and coagulopathic isoforms have also been described^55,56^. Interestingly, the neurotoxic, cytotoxic and some of the coagulopathic effects caused by these PLA_2_s are based upon the hydrolysis of phospholipids, either present in cellular membranes or as free phospholipids in plasma^22,57^. In contrast, the mechanism of action of a few anticoagulant elapid PLA_2_s is related to the binding and inhibition of coagulation factors^57^. Group II PLA_2_s can be either enzymatically active or inactive depending on whether key residues at the catalytic site and the calcium-binding loop necessary for catalysis are mutated or not^55^. The enzymatically active PLA_2_s disrupt the membrane by hydrolysis of membrane phospholipids. In contrast, the enzymatically inactive toxins, known as PLA_2_ homologs, exert their effects via perturbation of the plasma membrane in the absence of phospholipid hydrolysis^22,55,58,59^. A mechanism of membrane disruption by catalytically-inactive Lys49 PLA_2_ homologs has been proposed in which two distinct molecular regions are involved, initially in the binding to the membrane and then in the penetration and disruption of membrane integrity^60,61^ (see Supplementary Fig. 1).

A synergistic effect of catalytically active and inactive PLA_2_s for inducing myotoxicity has been described^62^. Group II PLA_2_s include neurotoxic and cytotoxic PLA_2_s, the latter being able to affect skeletal muscle cells by either binding to specific protein receptors or low-affinity lipid domains in the plasma membrane^22,58^. In addition, cytotoxic PLA_2_s and PLA_2_ homologs are cytotoxic to a variety of cell types in culture^35,63^.

The destabilisation of the lipid bilayer makes the membrane more permeable to ions, thereby losing its membrane potential (becoming depolarised) and allowing a large influx of Ca^2+^ from the extracellular medium^21,22,64,65^. The uncontrolled influx of calcium causes calcium overload in affected cell types (i.e., myocytes, nerve terminals) and induces more stress on the weakened cellular membrane and mitochondrial dysfunction, resulting in decreases in ATP production, which in turn may result in necrosis^21,22^. In addition, such an increase in cytosolic calcium concentration affects the cytoskeleton, inducing hypercontraction in muscle cells^66^. The effect of cytotoxic PLA_2_s on cells varies depending on toxin concentration thresholds. For example, at different concentrations, a Lys49 PLA_2_ homolog devoid of enzymatic activity induces necrosis, apoptosis or cell proliferation in a lymphoblastoid cell line, and these effects are related to variable degrees of alterations in calcium homeostasis^67,68^.

### Endocytosis followed by lysosomal degradation by β-defensin-like toxins

β-defensin-like toxins form another group of cytotoxins. These toxins are small (4-5 kDa) non-enzymatic homologs of endogenous cell-penetrating β-defensins and are only found in the venoms of certain pitvipers (subfamily Crotalinae)^69,70^. β-defensin-like toxins penetrate plasma membranes through endocytosis via their high net-positive charge that facilitates interactions with negatively charged cell surfaces. Upon binding their targets (i.e., ion channels and proteoglycans), β-defensin-like toxins are internalised and accumulate in lysosomal vesicles. This accumulation results in disruption of the lysosome membrane and subsequent lysosome lysis, which in turn promotes caspase activity, triggering apoptosis^71,72^. In addition to their cell-penetrating properties, these toxins interact with voltage-gated ion channels, altering their osmotic balance and inducing alterations in membrane potential^37,72,73^. The most extensively studied snake β-defensin-like toxins are crotamine (found in *Crotalus durissus*) and myotoxin-α (isolated from *Crotalus viridis viridis*)^69^.

These toxins are generally named “myotoxins” because they induce muscle contracture and morphological alterations in myofibres^74^. However, other studies have shown that the cytoplasmic accumulation of these toxins occurs across various cell types^75^. Depending upon whether this internalisation in other cell types results in cytotoxicity, the name ‘myotoxin’ might be inaccurate, but this issue remains to be investigated. In the meantime, we suggest naming this class of cytotoxins ‘β-defensin-like toxins’.

## Indirect cytotoxic effects by toxins that degrade the extracellular matrix

The extracellular matrix (ECM) is a macromolecular structure made up of the interstitial matrix and the basement membrane. The interstitial matrix consists of several types of collagens, as well as fibronectin, various proteoglycans, and hyaluronic acid, and plays critical roles in the homeostasis of tissues^40,76^. The basement membrane consists of laminin, collagen type IV and VI, perlecan and nidogen, in addition to other minor components. It surrounds a variety of cell types, forming a connection between these cells and the interstitial matrix^77^. The basement membrane has multiple functions, such as providing structural support to capillary endothelial cells and many other cell types, acting as a filtration barrier, storing growth factors, preventing cells and larger molecules from passing through, and organising the tissue architecture. This barrier function is also observed in the capillaries, where the basement membrane prevents the extravasation of white blood cells until these are activated in inflammation by signalling molecules such as cytokines^78^. The basement membrane in the microvasculature also provides mechanical support to withstand the biophysical forces that generally operate in the circulation^17^. Because of its crucial role, the ECM is a primary target for various tissue-damaging components in snake venoms^79,80^.

### Degradation of key components of the ECM by snake venom metalloproteinases

Snake venom metalloproteinases (SVMPs) are found in all venomous snake families, being more abundant in species of the family Viperidae^70,81^. SVMPs are divided into three major classes (named P-I to P-III) based on their domain structure. Toxins of the class P-I (~20–30 kDa) only contain the metalloproteinase domain, while the second class, P-II (~20–60 kDa), carries an additional disintegrin domain which may remain intact or be proteolytically liberated. Finally, the P-IIIs (~60–100 kDa) have a disintegrin-like domain and a cysteine-rich domain in addition to the catalytic domain and can also be post-translationally modified in various ways^82^. The metalloproteinase domain is capable of enzymatic degradation of key components of the ECM, thereby destabilising the interactions between cells and the basement membranes and disrupting the overall structural arrangement of the ECM^80^. The degradation of the basement membrane by SVMPs affects a variety of cell types, including endothelial cells, skeletal muscle cells, keratinocytes and kidney cells^23,27,80^. The hydrolysis of the basement membrane that surrounds endothelial cells in capillaries results in the weakening of the capillary wall, followed by the distention and eventual disruption of endothelial cells, leading to extravasation^17,79^. Thus, SVMPs are cytotoxic to endothelial cells in vivo through this indirect mechanism in which cell death occurs by the action of mechanical forces operating in the circulation secondarily to the weakening of the basement membrane as a consequence of SVMP-induced hydrolysis^17^. The hydrolysis and subsequent disruption of the ECM organisation further facilitate the diffusion of toxins into the circulation, giving SVMPs a spreading factor-like effect^80,83^. The additional domains present in P-II and P-III SVMPs are likely to bind to targets in the vasculature and in the tissue, thus directing these enzymes to specific sites and contributing to their toxicity. It has been proposed that such targeting enables these enzymes to have a more potent haemorrhagic activity as compared to P-I SVMPs^79,84,85^.

By causing microvessel disruption and haemorrhage, SVMPs exert an indirect cytotoxic activity. Haemorrhage affects blood perfusion to tissues, thus generating ischemia, which affects the viability of cells. It has been shown that haemorrhagic SVMPs induce skeletal muscle necrosis through this indirect mechanism of cytotoxicity^36,86^. Moreover, hydrolysis of BM in glomeruli contributes to the renal pathology characteristic of envenomings by viperid snakes^87^. Furthermore, beyond the action of haemorrhagic SVMPs on the basement membrane, both haemorrhagic and nonhaemorrhagic SVMPs degrade components of the interstitial matrix, thus contributing to the overall disorganisation of the ECM^88^. In addition to the general tissue disorganisation, the hydrolysis of ECM components by SVMPs may result in the release of growth factors stored in the matrix and in the generation of biologically active ECM fragments, which participate in inflammatory reactions and may further contribute to tissue damage^80^.

### Degradation of hyaluronic acid by hyaluronidases

Hyaluronidases are low-abundant enzymes ( ~ 52-55 kDa) found in elapid, viperid and some colubrid venoms^54,70,89^. Their main activity is the hydrolysis of hyaluronic acid, one of the key components of the ECM, and therefore contributes to ECM degradation^90,91^. The loss of the ECM structure integrity promotes the diffusion of (other) toxins, giving hyaluronidase its name as a ‘spreading factor’. Therefore, hyaluronidases play a dual role in envenoming: they degrade hyaluronic acid, thus contributing to ECM disorganisation, and they likely contribute to the local and systemic spreading of venom toxins, potentiating the tissue-damaging effect of cytotoxins and SVMPs^92–94^. Table 1 summarises the main types of tissue-damaging toxins in snake venoms, their targets and their main pathological and pathophysiological effects.

**Table 1:** Overview of effects of tissue-damaging toxins in snake venoms, which exert their actions in vivo, including directly cytotoxic toxins and enzymes that degrade the ECM

Directly cytotoxic toxins		
Toxin class	Main targets	Pathological and pathophysiological consequences
3FTxs	Skeletal muscle	Myonecrosis (local and systemic)
	Skin	Dermonecrosis
	Cardiac muscle	Cardiotoxicity
	Erythrocytes	Intravascular haemolysis
	Other cell types	Cytotoxicity
PLA_2_s	Skeletal muscle	Myonecrosis (local and systemic)
		Acute kidney injury (through the toxic action of myoglobin)
	Skin	Dermonecrosis
	Kidney	Acute kidney injury (secondary to cytotoxic action on renal cells)
	Erythrocytes	Intravascular haemolysis
	Other cell types	Cytotoxicity
β-defensin-like toxins	Skeletal muscle	Contracture
		Myonecrosis
Enzymes that degrade ECM		
Toxin class	Main targets	Pathological and pathophysiological consequences
SVMPs	Basement membrane in capillary vessels	Disruption of the integrity of microvessels with extravasation (haemorrhage)
		Ischemia secondary to haemorrhage and reduction of blood flow in various tissues (i.e., skeletal muscle, kidney)
	Skin	Blister formation secondary to cleavage of proteins in the dermal-epidermal junction
		Dermonecrosis
	Proteins in ECM	Widespread degradation of proteins in ECM, with loss of tissue organisation and spreading of venom components
Hyaluronidases	Hyaluronic acid in ECM	Disorganisation of the ECM
		Spreading of venom components

## Venom components induce an inflammatory response that may contribute to tissue damage

Injection of tissue-damaging venom components in tissues, especially PLA_2_s and SVMPs, promotes a complex inflammatory response associated with the synthesis and secretion of a plethora of mediators and a prominent inflammatory infiltrate of neutrophils and macrophages^20^. These processes contribute to reparative and regenerative tissue processes but may also have harmful consequences in the tissues. One outcome of this process is the generation of reactive oxygen species, resulting in oxidative stress and consequent tissue alterations^95,96^. Moreover, the toxic effects of PLA_2_s and SVMPs in tissues result in the generation of ‘damage-associated molecular patterns’, which stimulate innate immunity and expand the inflammatory response^97,98^. ATP released from damaged cells may also amplify the cell-damaging effect by acting on purinergic receptors in neighbouring cells^99^. Thus, venom-induced tissue damage is likely to be mediated by the direct action of venom components discussed above, as well as by endogenous processes whose role in the overall venom-induced pathology remains to be determined.

## The clinical consequences of tissue-damaging activities of venoms

The mechanisms of tissue damage induced by venom toxins discussed above have direct implications in the pathology and pathophysiology of snakebite envenoming. These clinical manifestations can be roughly divided into local and systemic effects.

### Local tissue-damaging effects

Snake venoms are generally delivered at the bite site by the subcutaneous or intramuscular injection routes. Once in the tissue, various types of toxins exert tissue damage of rapid onset, especially in venoms of viperid species and some elapid species, such as the spitting cobras^1,3^. In the case of viperid venoms, cytotoxic PLA_2_s induce acute skeletal muscle necrosis as a consequence of the direct effect of these toxins on the integrity of the plasma membrane of muscle cells, with the consequent influx of calcium ions, causing a series of intracellular degenerative events^21,22^. Cytotoxic PLA_2_s also affect lymphatic vessels^100^, thus contributing to the accumulation of fluid in the tissue (oedema). Cytotoxic venoms of spitting cobras (*Naja* spp.) likely cause muscle damage by the combined action of cytotoxic PLA_2_s and cytotoxic 3FTx and induce cutaneous necrosis associated with drastic alterations in the various layers of the skin^25,101^. A unique clinical manifestation caused by venom spitting of certain cobras is observed as painful ophthalmic lesions, which are the result of the direct action of cytotoxic 3FTxs and PLA_2_s in victims that have been spat in the eyes^33,102^. Cytotoxic cobra venoms and viperid venoms also induce the formation of skin blisters, which, in the case of the latter, is a consequence of the action of SVMPs on the dermal-epidermal interface, resulting in the separation of dermis and epidermis^27,103^. In addition to damage to muscle and skin, tissue-damaging toxins also affect nerves and arteries^104,105^. As a result of the extravasation of blood and plasma, viperid envenomings are often associated with an increase in intracompartmental pressure in some muscle compartments, which affects arterial perfusion and might end up in ischemic necrosis associated with compartmental syndrome^1^.

Envenomings by viperid species are generally accompanied by widespread degradation of ECM, mediated by SVMPs and hyaluronidases. The hydrolysis of basement membrane components results in microvessel disruption and haemorrhage^1,79^. As a consequence, blood perfusion is impaired, and ischemia ensues. This effect contributes to skeletal muscle necrosis^86^ and precludes the regeneration of muscle tissue^106^. ECM degradation also results in the disorganisation of the matrix^80^, thus affecting a variety of processes that depend on matrix integrity. Such degradation also contributes to the diffusion of venom components locally and systemically.

### Systemic tissue-damaging effects

Snake venoms are distributed systemically through lymphatic and blood vessels, thus reaching diverse organs where they cause harmful effects. In the case of venoms of some rattlesnakes, Australian elapids and sea snakes, cytotoxic PLA_2_s induce systemic muscle necrosis, i.e., rhabdomyolysis^3,107,108^. As a consequence, there is a massive release of cytosolic muscle components, such as creatine kinase and myoglobin, into the circulation. Myoglobin, in turn, contributes to the acute kidney injury characteristic of these envenomings^1,109^. Moreover, an increase in potassium concentration in the blood (hyperkalaemia), as a result of rhabdomyolysis, may affect cardiac function^108^. In some venoms, cytotoxic components cause intravascular haemolysis by disrupting the integrity of the plasma membrane of erythrocytes^110,111^. By damaging the integrity of blood vessels in various organs, haemorrhagic SVMPs cause systemic bleeding, which is potentiated by the action of haemotoxic components that affect haemostasis^15^. This impairs blood perfusion to organs, which might lead to cardiovascular shock and multiple organ failure.

**Fig. 4:**
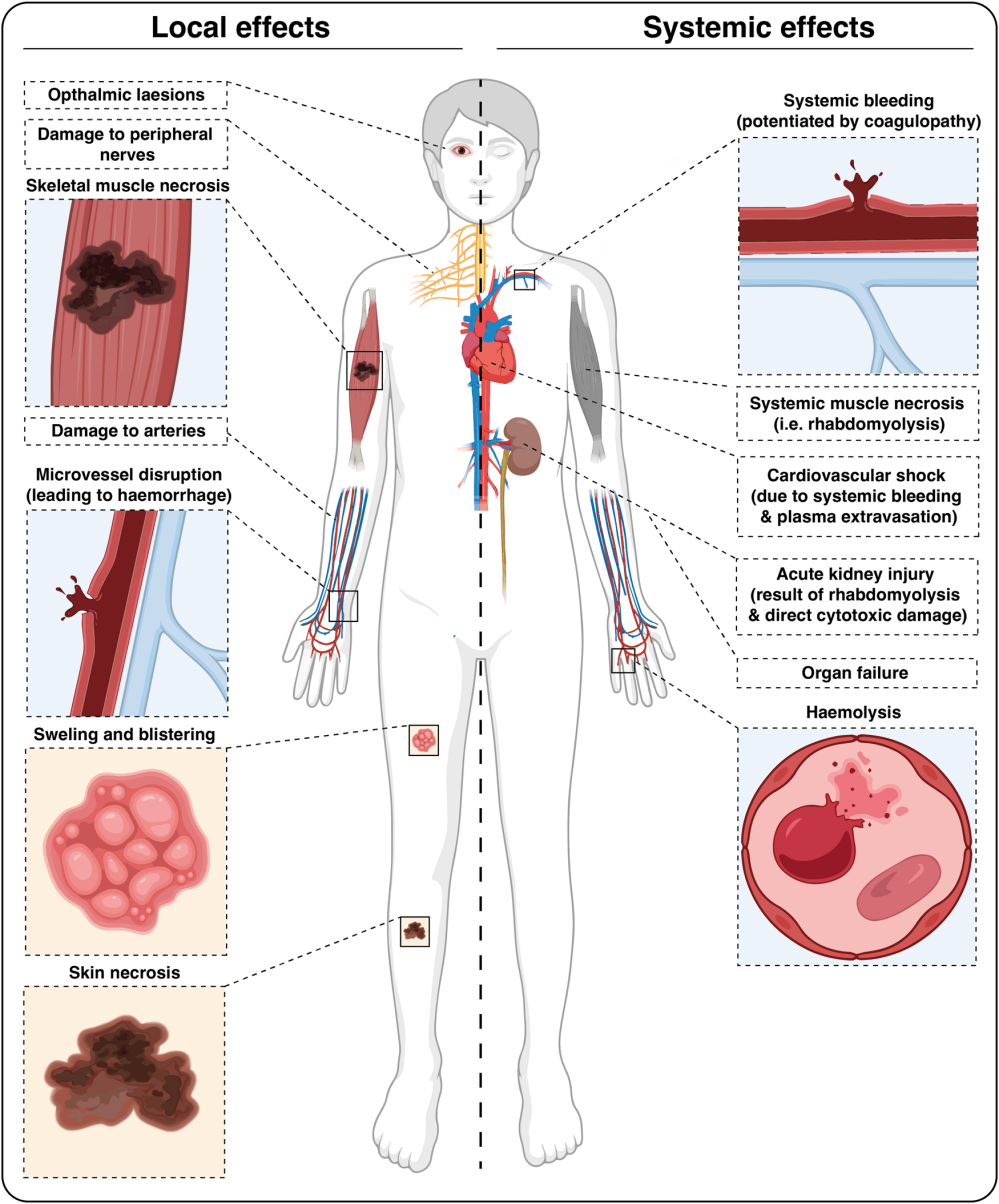
Overview of the tissue-damaging activities of snake venom toxins on various body systems. Snake venoms may cause a wide range of effects in the human body and depend on the composition of the venom. The observed effects can be local and systemic. The image was created via www.biorender.com (with permission).

Cytotoxins and ECM degrading enzymes may have a direct impact on the kidneys, causing acute kidney injury through a variety of mechanisms that include degradation of basement membrane in glomeruli, direct cytotoxicity on renal tubular cells, hypoperfusion associated with systemic bleeding, and the toxic effect of myoglobin and haemoglobin, released as a consequence of rhabdomyolysis and haemolysis, respectively, on renal tubular cells^112^. Likewise, venom cytotoxic components are likely to affect other tissues and cells, thus contributing to the overall pathophysiology of envenomings, including myocardial damage^113,114^. Figure 4 summarizes the most important local and systemic effects induced by snake venoms.

## Mechanisms of cytotoxicity observed in vitro but with unknown impact on in vivo envenoming

Studies on the action of venoms and toxins on cells in vitro have revealed mechanisms of cytotoxic effects that provide valuable information on the action of these toxins and may shed light on more general aspects of cellular pathology which could be applied to the understanding of other diseases. However, the actual in vivo and clinical implications in snakebite envenomings of several of these in vitro experimental observations remain unknown. These mechanisms will be described as they illustrate possible ways through which snake toxins affect the viability of cells and might illuminate the design of future revealing in vivo studies.

### Apoptosis induced by anoikis

A variety of venom components have been shown to induce apoptosis of several cell lines in culture. One of the main mechanisms of apoptosis in vitro occurs via anoikis, resulting from the detachment of cells from their substrate^115,116^. Upon loss of cell-cell and cell-ECM adhesions, the inhibitory effects on the cell death pathways are lifted, which causes the cell to undergo apoptosis^117,118^. Although the mechanisms of anoikis are poorly understood, it is thought that the disruption of focal contacts is the primary signal for activation of the extrinsic apoptotic pathway^117,119,120^. Cell detachment has been described as a consequence of the action of SVMPs on endothelial cells in culture^116,121^. SVMPs degrade the matrix upon which cells are adhered or might be able to cleave integrins in these cells, which interact with matrix components, thus inducing the detachment of cells. However, SVMPs also induce apoptosis by mechanisms other than anoikis, probably related to interaction with integrins and related molecules^122^. Further, some SVMPs induce apoptosis in non-adherent cell lines, suggesting that mechanisms other than anoikis might be at play^123^.

Disintegrins are venom components predominately derived from the cleavage of P-II SVMPs^82,124^. They bind to integrins and induce a variety of effects on cells in vitro^125^. The binding of disintegrins leads to a loss of cell-cell and cell-ECM adhesions, which may trigger the anoikis pathway^125^. Another class of proteins that are thought to affect cells in vitro are C-type lectin family members, which are found in viperid, elapid and colubrid venoms^70,81^. A diverse range of effects is described for these toxins, including cytotoxicity on various cancer cell lines, although the exact mechanisms have not yet been elucidated^126–128^.

### ATP release secondary to toxin-induced cytotoxicity leads to activation of cell death pathways

The direct cytotoxic action of PLA_2_s results in the release of cytosolic molecules, including ATP, to the extracellular environment^99,129^. It has been shown that this nucleotide interacts with purinergic receptors in myogenic cells, acting as a ‘danger signal’ and spreading cell damage and inflammation, evidenced by the increase in cytosolic calcium^99,130,131^. This effect was reduced by apyrase, an enzyme that degrades ATP, thus underscoring the role of this nucleotide in the effect^99^. Moreover, a cytotoxic Lys49 PLA_2_ homolog induces cell death in macrophages in culture by a mechanism related to ATP release and action on purinergic receptors^132^.

### Apoptosis induced through ROS generation by L-amino acid oxidases (LAAOs)

Reactive oxygen species (ROS) are produced as by-products during aerobic metabolism in cells. Although low ROS levels are intrinsically associated with normal cellular functioning, the accumulation of ROS (e.g., hydrogen peroxide) is suggested to be the primary inducer of oxidative stress^133^. This may affect cellular processes by denaturing enzymes, disturbing the cell membrane, and inducing DNA damage, which could ultimately result in cell death^134^. Hydrogen peroxide is generated by the action of L-amino acid oxidase (LAAO) present in snake venoms^135^. LAAOs are enzymatic flavoproteins with a molecular weight of 50–70 kDa, which are found in most snake species^70,136^. LAAOs catalyse the oxidative deamination of L-amino acids and produce hydrogen peroxide. LAAOs may induce apoptosis and necrosis depending on their concentration^137–139^.

Several isoforms of LAAO, isolated from viperid and elapid snake venoms, have been shown to induce apoptosis of several cell types in culture, including cancer cell lines. In general, cytotoxicity is due to the generation of H_2_O_2_ as a consequence of the enzymatic activity of LAAOs^137,140–143^. Such oxidative stress induces apoptosis by the extrinsic and intrinsic pathways, with increases in caspases and other proapoptotic enzymes, degradation of DNA and loss of mitochondrial membrane potential^141,144^. Interestingly, an LAAO can induce various forms of cell death, as shown in an enzyme from the venom of *Bothrops atrox*, which induces apoptosis, autophagy and necrosis in a keratinocyte cell line^138^.

### Activation of various cell death pathways by toxins that induce direct cytotoxicity in vivo

**Fig. 5:**
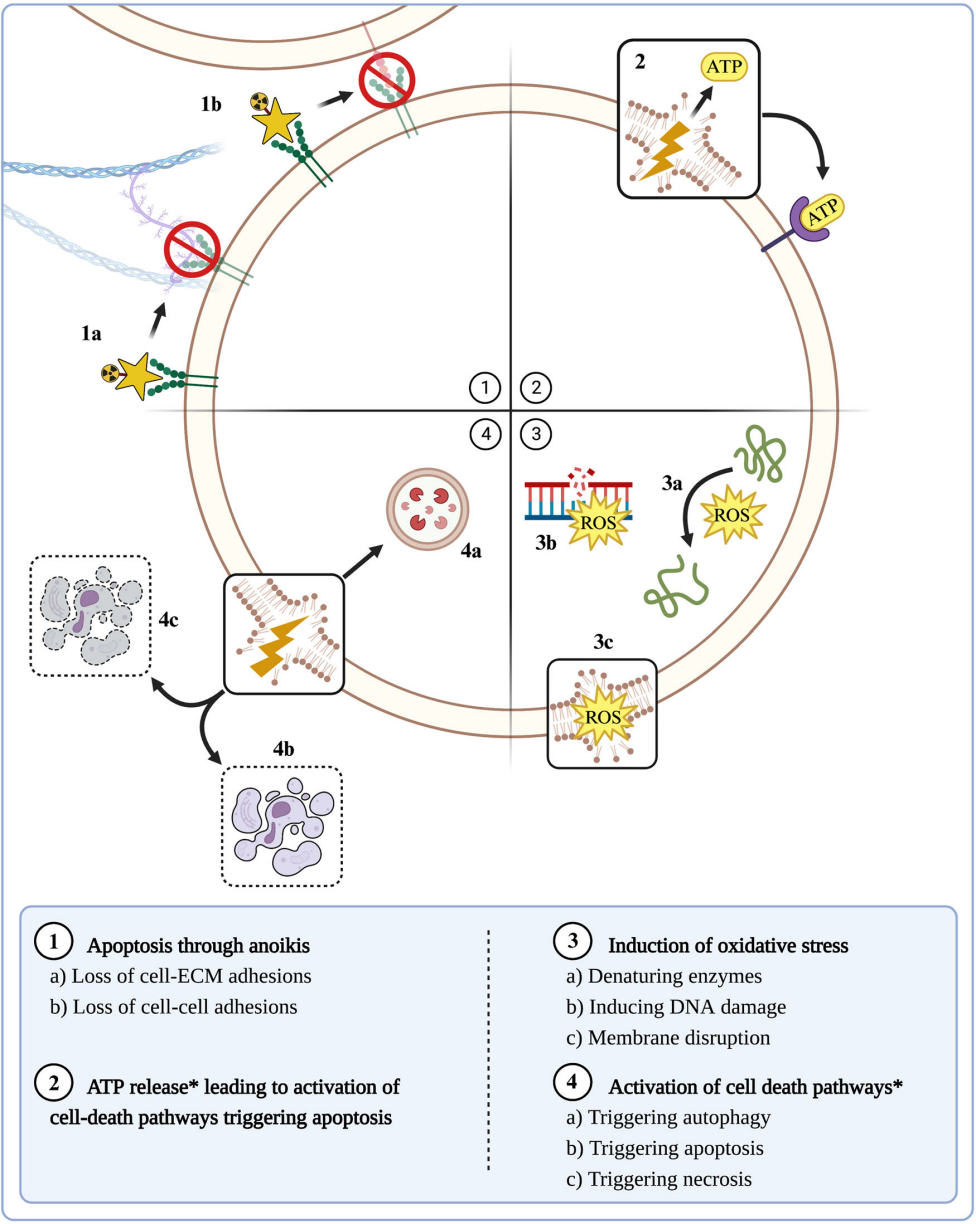
Schematic overview representing the various mechanisms of cytotoxicity observed in vitro. (1) Apoptosis through anoikis by SVMPs, disintegrins (and possibly C-type lectins). (2) ATP release leads to the activation of cell-death pathways and, thereby, triggering apoptosis by cytotoxic PLA_2_s. (3) Apoptosis triggered by ROS production by LAAOs. (4) Activation of various cell death pathways by cytotoxic 3FTxs and cytotoxic PLA_2_s. Asterisk depicts those mechanisms which are secondary to direct cytotoxicity. The image was created via www.biorender.com (with permission).

Cytotoxic 3FTxs and PLA_2_s induce necrosis in vivo as well as in cells in culture, as described above. It has been observed that these cytotoxic components also induce apoptosis in various cell lines, including cancer cells, in vitro. A variety of cytotoxic 3FTxs trigger apoptotic pathways^145–147^, as well as autophagy^148^. A cytotoxic Lys49 PLA_2_ homolog was shown to induce necrosis, apoptosis, and proliferation in a lymphoblastoid cell line, the outcome of which is dependent on toxin concentration^67^. Therefore, it is likely that these toxins induce varying cellular responses depending on their concentration. In vivo, this might translate into different cell death thresholds in tissues, whereby necrosis may predominate in regions of high toxin concentration. In contrast, apoptosis and autophagy may occur in areas of lower toxin concentration, and even proliferative or stimulatory effects may be seen in areas of even lower concentrations, in a complex gradient that might vary over time^149^. Figure 5 summarises the various mechanisms of cytotoxicity induced by venom components which have been described in vitro.

The cytotoxic mechanisms described in this section highlight the complexity of the actions of venom components in cells. Even though these phenomena have been studied in cell culture conditions in vitro, they may shed light on possible pathogenic mechanisms that operate in vivo. It is necessary to explore the occurrence of these cell death mechanisms in vivo and to assess their relevance in the clinical manifestations of envenomings. Thus, it would be relevant to determine whether the various types of cell death described in cell culture conditions also occur in tissues affected by snake venom toxins in vivo. Moreover, the study of the mechanisms involved in cytotoxicity may shed light on potential new therapeutic options for snakebite patients.

## The challenge of developing effective therapies for venom-induced tissue damage

Current snakebite treatments consist of the administration of antibodies derived from serum or plasma that are produced by hyper-immunisation of large animals (primarily equines or ovines) with snake venoms^150,151^. Animal-derived antivenoms are generally effective in the abrogation of systemic, life-threatening effects of snake venoms^1,3^. However, their efficacy in neutralising venom-induced local tissue damage is somewhat limited^25,152,153^. This is due to a number of factors, especially the rapid onset of these pathological effects, associated with the frequent delay in antivenom administration. Since antivenoms induce adverse reactions in a percentage of patients, they should be administered by health staff in hospitals and clinics, which, quite often, are located far from the region where the snakebite occurred. In addition, there are countries and regions within countries where the availability and accessibility of antivenoms is limited^1,154,155^. Moreover, some toxins responsible for tissue damage, such as cytotoxic 3FTxs and PLA_2_s, are poorly immunogenic and consequently, the antibody titres against these toxins in antivenoms tend to be low^156,157^. Therefore, there is an urgent need to develop novel therapeutic strategies to confront this aspect of snakebite envenoming. To address these problems, recent studies have been focusing on the development of alternative snakebite treatments. Combining the growing body of scientific knowledge of the (tissue-damaging) snake venoms with modern bioanalytical chemistry techniques and biotechnological approaches allows for the exploration of novel inhibitory compounds.

A highly promising approach is based on the repurposing of enzyme inhibitors that have been developed for a variety of diseases in which endogenous PLA_2_s and SVMPs play a crucial role. Owing to the structural similarities of these endogenous enzymes and venom enzymes, some of these inhibitors, which have already gone through clinical trials for other diseases, are particularly effective in the inhibition of venom PLA_2_s and SVMPs. The most promising group of synthetic PLA_2_-inhibitors are the indoles (i.e., LY315920 Varespladib and LY333013 methyl Varespladib). These inhibitors were proven to be effective at protecting against various PLA_2_-mediated pathophysiological alterations, including inflammatory diseases^158–160^. They have also been tested in animal models of snake envenoming, and they inhibit venom toxicity, including lethality-inducing neurotoxic effects and coagulopathy, as well as tissue-damaging effects, i.e., inhibition of myonecrosis and dermonecrosis^161–167^. Interestingly, LY333013 is administered by the oral route, therefore representing a convenient option for therapy in the field, owing to the good safety profile of this compound. A clinical trial is underway to test this oral inhibitor in snakebite envenoming^168^. It is necessary to demonstrate whether an orally administered drug would be able to reach the tissue affected by the venom in a timely manner so as to prevent or reduce tissue damage.

As with PLA_2_s, there is a wide array of endogenous metalloproteinases that have a variety of physiological roles and have been implicated in human disease, which have, therefore, been intensively targeted to find inhibitory candidates^162,169,170^. As the effects of SVMPs are predominantly zinc-dependent, inhibitors that interact with the Zn^2+^ in such a way that the catalytic effect of the toxins is abrogated have been explored. Currently, two classes of SVMP inhibitors have been studied as candidates for SVMPs, both with a distinct mode of action. Peptidomimetic inhibitors (e.g., the matrix metalloproteinase inhibitors Batimastat, Marimastat, Prinomastat) directly bind the zinc ion in the binding pocket of the protein while having an affinity for the catalytic site^167,171–174^, whereas the metal chelators (e.g., Dimercaprol, DMPS, EDTA) work by chelating the zinc moiety required for catalysis^167,172,174,175^. Similar to Varespladib, some of these drugs show exciting potential for snakebite by preventing pathology in animal models of envenoming and because the oral route can be used in some of them^162,167,175^. Combination therapies of PLA_2_s and SVMP inhibitors are currently being investigated and have already shown promising potential in inhibiting tissue-damaging activities^167,176^.

The search for inhibitors against other groups of toxins responsible for tissue damage, such as hyaluronidases, LAAOs and other tissue-damaging toxins, has been more limited^162,169^. Although hyaluronidase inhibitors have been described, these compounds showed inhibitory concentrations (IC_50_) in the micro- and millimolar range, raising the question of whether these can be considered promising candidates for clinical studies^93,94,177^. One advantage of enzyme inhibitors as compared to antibodies is that their spectrum of inhibition is broader since they are generally directed against the active sites of enzymes, which are similar for each group of enzymes, in contrast to the antigenic variability of toxic enzymes from different venoms^162,169,178^.

Other promising therapies include recombinant- and monoclonal antibodies or antibody fragments^169^. Recombinant antibodies can be developed against poorly immunogenic toxins responsible for tissue damage^179^. In addition, antibodies can be designed in formats that facilitate the neutralisation of these toxins in the tissues^180^. For example, low molecular mass recombinant antibodies, such as nanobodies and similar formats, have a higher volume of distribution than whole IgG molecules, having the capacity to reach tissue compartments and neutralise toxins in the tissues.

Synthetic toxin inhibitors include aptamers (i.e., single-stranded DNA- or RNA-oligonucleotides), synthetic peptides and synthetic nanoparticles, all of which have been selected for their capacity to bind and neutralise toxins^181–184^. Another promising strategy comprises a decoy receptor approach using mimotopes (i.e., peptides mimicking the structure of the subunits of acetylcholine binding protein), thereby preventing the binding of 3FTx to native (acetylcholine) receptors^185^. The binding of the mimotopes to these toxins would prevent their interaction with native receptors, thereby neutralising their effect. Although this approach has been solely explored on neurotoxic 3FTxs, other studies have shown the affinity of cytotoxic 3FTxs for the acetylcholine-binding protein (i.e., a structural homologue of the binding domain on the acetylcholine receptor)^186,187^. Therefore, the possibility of being capable of neutralising the cytotoxic effects should not be ruled out. Identification of the sequences of receptors that bind these toxins may eventually lead to the synthesis of a wide array of these decoy receptors that may block the activity of cytotoxic 3FTxs^162^. In turn, nanoparticles of various chemical compositions can be designed with the ability to inhibit cytotoxic 3FTxs, PLA_2_s and SVMPs and have been shown to be effective against the venoms of cytotoxic *Naja* spp^184,188^.

In addition to antibodies of variable formats, there is a large body of research focusing on the development of venom inhibitors from a variety of natural sources^162^. Natural inhibitors have been described in plants and animals^189–191^. For example, endogenous PLA_2_-inhibitors present in the plasma of some animals (e.g., snakes and opossums) have been proposed as candidates^190,192–195^. These protein inhibitors are part of the innate immune system of these animals that are used to counteract the effect of snake venoms and form complexes with the PLA_2_ toxins^192^. Likewise, inhibitors of SVMPs have been characterised from the blood of mammals and snakes^191,195^. The inhibitory mechanism of these endogenous inhibitors remains largely unknown, but in some cases, they form macromolecular complexes with toxins^162,190,195^.

Abundant literature exists on the plant-derived extracts capable of inhibiting the effects of PLA_2_s and SVMPs. However, less is known about the structural detail of their inhibitory effects, primarily since less research has focused on the isolation and characterisation of isolated plant-derived compounds^162,169,196–199^. Table 2 presents an overview of several novel inhibitor candidates against tissue-damaging toxins of snake venoms.

**Table 2:** Overview of novel therapeutic candidates for inhibition of tissue-damaging compounds in snake venoms

Toxin class	Therapeutic candidate	Examples	References
Major toxin classes			
Cytotoxic 3FTxs	Aptamers	Aptamers developed against α-bungarotoxin^*^	^181,182^
	Synthetic peptides	Peptide inhibitors against α-cobratoxin^*^	^183^
	Nanoparticles	Nontoxic hydrogel copolymer nanoparticles	^184^
	Mimotopes (i.e., decoy receptors)	Recombinant nAChR mimics	^185,186,200,201^
Cytotoxic PLA_2_s	Nanoparticles	Nontoxic hydrogel copolymer nanoparticles	^184,202^
	Polyanions	Suramin; Heparin	^203–207^
	Animal-derived compounds	PLA_2_ inhibitors from mammals and snakes	^190,192,194,208^
	Plant-derived compounds	Alkaloids (e.g., aristolochic acid); rosmarinic acid	^196–199,209–213^
	Small molecule inhibitors	LY315920 Varespladib; LY333013 methyl Varespladib	^161,164–167^
SVMPs	Nanoparticles	Synthetic polymer nanoparticles	^188,214^
	Animal-derived compounds	SVMP inhibitors isolated from mammals and snakes	^191,215,216^
	Plant-derived compounds	Flavonoids	^197,217,218^
	Small molecule inhibitors	Marimastat, batimastat, prinomastat	^167,171,172,174^
		Dimercaprol; DMPS; EDTA	^167,172,174^
Minor toxin classes			
Hyaluronidases	Plant-derived compounds	Alkaloids (e.g., aristolochic acid); flavonoids (e.g., quercetin);	^91,93,177,189,219^
	Polyanions	Heparin	^91,93,220^
β-defensins-like toxins	Currently none	-	-

^*^Inhibitors that were originally designed for neurotoxic 3FTxs but have also the potential to be used for inhibition of cytotoxic 3FTxs.

The neutralisation and inhibition of tissue-damaging toxins by antivenoms and novel therapeutic alternatives constitute a considerable challenge, mainly because these effects develop rapidly after venom injection, and some of their consequences are irreversible. Thus, delays in the administration of antivenoms and novel therapeutics preclude an effective inhibition. Therefore, ideally, these novel therapies would have to be administered in the field rapidly after a snakebite to counteract the action of tissue-damaging toxins before they exert their effects. In addition, these therapeutics should have a pharmacokinetic profile that ensures effective access to the tissues where venom is injected. These tasks demand not only the generation of effective therapeutics but also of effective injection devices and public health interventions aimed to guarantee their availability and accessibility in regions of high incidence of snakebites. Moreover, the search for therapeutic options aimed at improving the processes of tissue repair and regeneration once tissue damage has developed constitutes another avenue to reduce the sequelae inflicted by this severe aspect of snakebite envenoming.

## Concluding remarks and forward-looking perspective

Snakebite envenoming is a public health issue of high impact which is responsible for causing mortality and long-term morbidity. The clinical syndromes can be broadly categorised into neurotoxicity, haemotoxicity and tissue-damaging effects, of which the latter is the leading cause of snakebite morbidity. Traditionally, some toxin subclasses were classified based on the tissues that they predominantly affect (e.g., cardiotoxins, myotoxins). Although the origin and reasoning of the latter classification method are apparent, this classification oversimplifies the complexity of these compounds and does not consider the mechanistic aspects of these toxins. This review provides an alternative classification for tissue-damaging toxins based on molecular mechanisms of action, including cytotoxicity and ECM degradation. In addition, it discusses the pathological and pathophysiological effects of tissue-damaging toxins and provides an overview of potential treatment strategies.

Many challenges remain in identifying the main toxins responsible for tissue damage in a variety of snake venoms, their mechanisms of action, and their clinical impact. Likewise, the in vivo manifestations of toxic mechanisms described in vitro, as well as the role of inflammatory and other endogenous processes in the pathogenesis of venom-induced tissue damage, remain to be investigated. An area in need of strengthening is the search for novel inhibitors of tissue-damaging toxins that would complement antivenoms in the therapy of these envenomings. Understanding the harmful actions of venoms in tissues should be integrated with the study of other pathologies that involve various forms of tissue damage (e.g., infectious diseases or cancer), thus highlighting general patterns of disease that pave the way for novel therapeutic alternatives.

### Supplementary information

Supplementary Information
