# Supplementary material for: Tissue damaging toxins in snake venoms: mechanisms of action, pathophysiology and treatment strategies
Source: Commun Biol. 2024 Mar 22;7:358. doi: 10.1038/s42003-024-06019-6 (PMC10960010; doi:10.1038/s42003-024-06019-6)
Supplement: Supplementary file 1 — Supplementary Information [file 42003_2024_6019_MOESM1_ESM.pdf]

Supplementary Table 1. Overview of the alternative names of several toxin classes with direct cytotoxic properties and a proposed name for the cytotoxic members of each protein family.

**Toxin class**	**Alternative names**	**Proposed name(s)**
Three-finger toxins (3FTxs)	TFTs (three-finger toxins)	Cytotoxic-3FTxs or neurotoxic 3FTxs
	TFPs (three-finger proteins)	
	Cytolysins	
	Membrane toxins	
	Membranotoxins	
	Direct lytic factors	
	Cytotoxins	
	Cardiotoxins	
PLA_2s	Myotoxins	Cytotoxic PLA_2s
β-defensin-like toxins	Small basic myotoxins	β-defensin-like toxins
	Small basic polypeptide myotoxins	

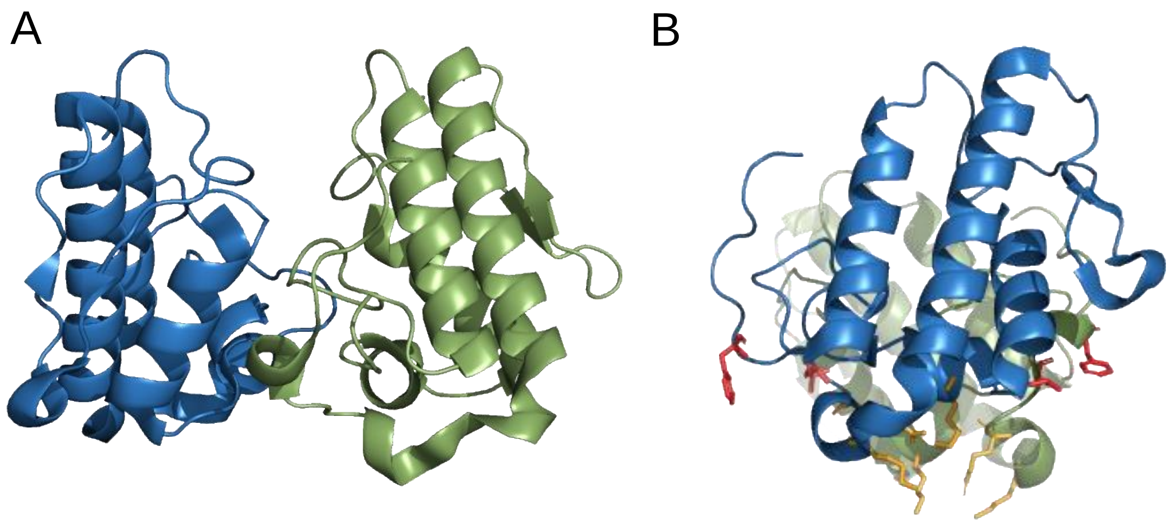

Supplementary Figure 1. Structure of a Lys49-PLA$_2$. (A) Cartoon representation of the three-dimensional structure of the dimer of Lys49-PLA$_2$ BthTx1 from *Bothrops jararacusu* determined by X ray crystallography (PBD 3hzd, Fernandes *et al.*, 2010, reference 61). Each monomer is colored in blue or green. (B) The cationic membrane-docking site composed by K20, K115 and R118 are represented by orange sticks, while the hydrophobic membrane-

disruption site composed by L121 and F125 are represented by red sticks in both PLA_2 monomers as described by Fernandes *et al*., 2014 (reference 60).
